# 1-Octyl-1*H*-benzimidazol-2(3*H*)-one

**DOI:** 10.1107/S1600536812013384

**Published:** 2012-04-04

**Authors:** Dounia Belaziz, Youssef Kandri Rodi, El Mokhtar Essassi, Lahcen El Ammari

**Affiliations:** a Laboratoire de Chimie Organique Appliquée, Université Sidi Mohamed Ben Abdallah, Faculté des Sciences et Techniques, Route d’immouzzer, BP 2202 Fès, Morocco; b Laboratoire de Chimie Organique Hétérocyclique URAC21, Faculté des Sciences, Université Mohammed V-Agdal, Av. Ibn Battouta, BP 1014, Rabat, Morocco; c Institute of Nanmaterials and Nanotechnology, MASCIR, Rabat, Morocco; d Laboratoire de Chimie du Solide Appliquée, Faculté des Sciences, Université Mohammed V-Agdal, Avenue Ibn Battouta, BP 1014, Rabat, Morocco

## Abstract

In the title compound, C_15_H_22_N_2_O, the octyl group adopts an all-*trans* conformation. In the crystal, mol­ecules form centrosymmetric dimers with an *R*_2_^2^(8) graph-set motif, linked by pairs of N—H⋯O hydrogen bonds. In addition, C—H⋯O contacts are observed.

## Related literature

For background to benzimidazol-2-one, see: Soderlind *et al.* (1999 ▶). For similar structures, see: Ouzidan *et al.* (2011 ▶); Kandri Rodi *et al.* (2011 ▶).
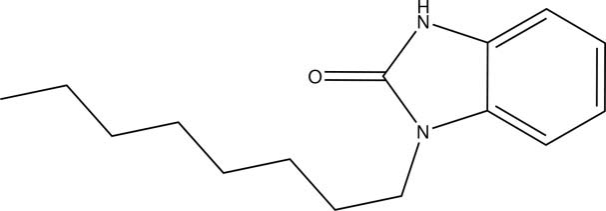


## Experimental

### 

#### Crystal data

C_15_H_22_N_2_O
*M* *_r_* = 246.35
Monoclinic, 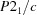

*a* = 14.8888 (18) Å
*b* = 5.8395 (6) Å
*c* = 16.6778 (19) Å
β = 91.448 (3)°
*V* = 1449.6 (3) Å^3^
*Z* = 4
Mo *K*α radiation
μ = 0.07 mm^−1^
*T* = 296 K
0.54 × 0.43 × 0.12 mm

#### Data collection

Bruker X8 APEX diffractometer
8760 measured reflections
3020 independent reflections
1971 reflections with *I* > 2σ(*I*)
*R*_int_ = 0.030

#### Refinement

*R*[*F* ^2^ > 2σ(*F* ^2^)] = 0.047
*wR*(*F* ^2^) = 0.143
*S* = 1.03
3020 reflections
164 parameters
H-atom parameters constrained
Δρ_max_ = 0.18 e Å^−3^
Δρ_min_ = −0.14 e Å^−3^

### 

Data collection: *APEX2* (Bruker, 2005 ▶); cell refinement: *SAINT* (Bruker, 2005 ▶); data reduction: *SAINT*; program(s) used to solve structure: *SHELXS97* (Sheldrick, 2008 ▶); program(s) used to refine structure: *SHELXL97* (Sheldrick, 2008 ▶); molecular graphics: *ORTEP-3 for Windows* (Farrugia, 1997 ▶); software used to prepare material for publication: *PLATON* (Spek, 2009 ▶) and *publCIF* (Westrip, 2010 ▶).

## Supplementary Material

Crystal structure: contains datablock(s) I, global. DOI: 10.1107/S1600536812013384/bt5862sup1.cif

Structure factors: contains datablock(s) I. DOI: 10.1107/S1600536812013384/bt5862Isup2.hkl

Supplementary material file. DOI: 10.1107/S1600536812013384/bt5862Isup3.cml

Additional supplementary materials:  crystallographic information; 3D view; checkCIF report

## Figures and Tables

**Table 1:** Hydrogen-bond geometry (Å, °)

*D*—H⋯*A*	*D*—H	H⋯*A*	*D*⋯*A*	*D*—H⋯*A*
N1—H1⋯O1^i^	0.86	2.01	2.8257 (19)	159
C4—H4⋯O1^ii^	0.93	2.52	3.312 (2)	144

Symmetry codes: (i) 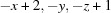; (ii) 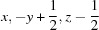.

**Fig. 1.**
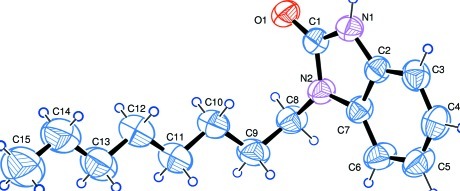
Molecular structure of the title compound with the atom-labelling scheme. Displacement ellipsoids are drawn at the 50% probability level. H atoms are represented as small circles.

**Fig. 2.**
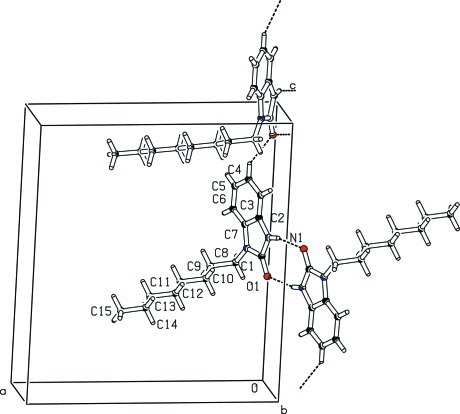
Molecule and its symmetry through the inversion center linked by hydrogen bonds and building dimers.

## supplementary crystallographic information

### Comment

Benzimidazol-2-one derivatives are useful heterocyclic building blocks and are prominent structural elements of compounds demonstrating a wide variety of pharmacological and biochemical properties (Soderlind *et al.*, 1999).

In this work, we have been able to react 1*H*-benzimidazol-2(3*H*)-one with 1-bromooctane in the presence of a catalytic quantity of tetra-n-butylammonium bromide under mild conditions to furnish the title compound (Scheme I).

The 1-octyl-1*H*-benzimidazol-2(3*H*)-one molecule structure is built up from fused six-and five-membered rings linked to C_8_H_17_ chain as shown in Fig.1. The fused-ring system is essentially planar, with a maximum deviation of 0.0045 (17) Å and 0.0080 (13) Å for C7 and N2 respectively. The dihedral angle between them does not exceed 1.20 (9)°. The octyl group is nearly perpendicular to the benzimidazole plane as indicated by the torsion angle of C1 N2 C8 C9 = -105.19(0.19)°. The structure of the title compound is similar to 1-nonyl-1*H*-benzimidazol-2(3*H*)-one (Ouzidan *et al.*, 2011) and 5-chloro-1-nonyl-1*H*-benzimidazol-2(3*H*)-one (Kandri Rodi *et al.*, 2011).

In the crystal, the molecules form centrosymmetric dimers linked by N—H···O hydrogen bonds with *R*_2_^2^(8) graph set motif.

### Experimental

To 1*H*-benzimidazol-2(3*H*)-one (0,2 g, 1,5 mmol), potassium carbonate (0.41 g, 3 mmol), and tetra-n-butylammonium bromide (0.1 g, 0.3 mmol) in DMF (15 ml) was added 1-bromooctane (0.3 ml, 1.8 mmol). Stirring was continued at room temperature for 6 h. The salt was removed by filtration and the filtrate concentrated under reduced pressure. The residue was separated by chromatography on a column of silica gel with ethyl acetate/hexane (1/2) as eluent. Colorless crystals were isolated when the solvent was allowed to evaporate.

### Refinement

H atoms were located in a difference map and treated as riding with N—H = 0.86 Å, C—H = 0.93 Å (aromatic), C—H = 0.97 Å (methylene) and C—H = 0.96 Å (methyl) with *U*_iso_(H) = 1.2 *U*_eq_ (aromatic, methylene) and *U*_iso_(H) = 1.5 *U*_eq_(methyl).

### Crystal data

**Table:**

C_15_H_22_N_2_O	*F*(000) = 536
*M**_r_* = 246.35	*D*_x_ = 1.129 Mg m^−^^3^
Monoclinic, *P*2_1_/*c*	Mo *K*α radiation, λ = 0.71073 Å
Hall symbol: -P 2ybc	Cell parameters from 3020 reflections
*a* = 14.8888 (18) Å	θ = 2.4–26.5°
*b* = 5.8395 (6) Å	µ = 0.07 mm^−^^1^
*c* = 16.6778 (19) Å	*T* = 296 K
β = 91.448 (3)°	Needle, colourless
*V* = 1449.6 (3) Å^3^	0.54 × 0.43 × 0.12 mm
*Z* = 4	

### Data collection

**Table:**

Bruker X8 APEX diffractometer	1971 reflections with *I* > 2σ(*I*)
Radiation source: fine-focus sealed tube	*R*_int_ = 0.030
Graphite monochromator	θ_max_ = 26.5°, θ_min_ = 2.4°
φ and ω scans	*h* = −18→18
8760 measured reflections	*k* = −5→7
3020 independent reflections	*l* = −19→20

### Refinement

**Table:**

Refinement on *F*^2^	Secondary atom site location: difference Fourier map
Least-squares matrix: full	Hydrogen site location: difference Fourier map
*R*[*F*^2^ > 2σ(*F*^2^)] = 0.047	H-atom parameters constrained
*wR*(*F*^2^) = 0.143	*w* = 1/[σ^2^(*F*_o_^2^) + (0.0608*P*)^2^ + 0.2949*P*] where *P* = (*F*_o_^2^ + 2*F*_c_^2^)/3
*S* = 1.03	(Δ/σ)_max_ < 0.001
3020 reflections	Δρ_max_ = 0.18 e Å^−^^3^
164 parameters	Δρ_min_ = −0.14 e Å^−^^3^
0 restraints	Extinction correction: *SHELXL97* (Sheldrick, 2008), Fc^*^=kFc[1+0.001xFc^2^λ^3^/sin(2θ)]^-1/4^
Primary atom site location: structure-invariant direct methods	Extinction coefficient: 0.010 (2)

### Special details

**Table:**

Geometry. All s.u.'s (except the s.u. in the dihedral angle between two l.s. planes) are estimated using the full covariance matrix. The cell s.u.'s are taken into account individually in the estimation of s.u.'s in distances, angles and torsion angles; correlations between s.u.'s in cell parameters are only used when they are defined by crystal symmetry. An approximate (isotropic) treatment of cell s.u.'s is used for estimating s.u.'s involving l.s. planes.
Refinement. Refinement of *F*^2^ against all reflections. The weighted *R*-factor *wR* and goodness of fit *S* are based on *F*^2^, conventional *R*-factors *R* are based on *F*, with *F* set to zero for negative *F*^2^. The threshold expression of *F*^2^ > 2σ(*F*^2^) is used only for calculating *R*-factors(gt) *etc*. and is not relevant to the choice of reflections for refinement. *R*-factors based on *F*^2^ are statistically about twice as large as those based on *F*, and *R*- factors based on all data will be even larger.

### Fractional atomic coordinates and isotropic or equivalent isotropic displacement parameters (Å^2^)

**Table:**

	*x*	*y*	*z*	*U*_iso_*/*U*_eq_	
O1	0.94346 (9)	0.1963 (2)	0.56368 (7)	0.0642 (4)	
N1	0.94058 (10)	0.1858 (2)	0.42412 (8)	0.0547 (4)	
H1	0.9690	0.0598	0.4166	0.066*	
N2	0.87525 (9)	0.4734 (2)	0.48345 (8)	0.0517 (4)	
C2	0.90690 (11)	0.3264 (3)	0.36345 (10)	0.0486 (4)	
C1	0.92224 (11)	0.2755 (3)	0.49722 (10)	0.0509 (4)	
C7	0.86541 (11)	0.5096 (3)	0.40093 (10)	0.0499 (4)	
C3	0.90920 (12)	0.3128 (3)	0.28082 (10)	0.0596 (5)	
H3	0.9368	0.1908	0.2554	0.072*	
C6	0.82615 (14)	0.6843 (3)	0.35775 (12)	0.0660 (5)	
H6	0.7991	0.8074	0.3830	0.079*	
C9	0.74157 (14)	0.6002 (4)	0.55652 (12)	0.0696 (6)	
H9A	0.7232	0.7133	0.5954	0.084*	
H9B	0.7117	0.6367	0.5058	0.084*	
C4	0.86880 (14)	0.4880 (4)	0.23756 (11)	0.0684 (6)	
H4	0.8688	0.4832	0.1818	0.082*	
C8	0.84181 (13)	0.6198 (3)	0.54643 (11)	0.0613 (5)	
H8A	0.8565	0.7776	0.5343	0.074*	
H8B	0.8721	0.5802	0.5967	0.074*	
C5	0.82845 (15)	0.6697 (4)	0.27511 (12)	0.0721 (6)	
H5	0.8022	0.7852	0.2441	0.087*	
C10	0.71026 (13)	0.3677 (4)	0.58346 (13)	0.0743 (6)	
H10A	0.7399	0.3306	0.6343	0.089*	
H10B	0.7282	0.2540	0.5446	0.089*	
C11	0.60948 (14)	0.3539 (4)	0.59315 (15)	0.0855 (7)	
H11A	0.5922	0.4626	0.6340	0.103*	
H11B	0.5800	0.3994	0.5431	0.103*	
C12	0.57598 (15)	0.1199 (4)	0.61598 (16)	0.0896 (7)	
H12B	0.6050	0.0758	0.6663	0.107*	
H12A	0.5944	0.0112	0.5756	0.107*	
C13	0.47537 (15)	0.1006 (5)	0.62485 (16)	0.0932 (8)	
H13A	0.4568	0.2077	0.6657	0.112*	
H13B	0.4460	0.1449	0.5746	0.112*	
C14	0.44383 (18)	−0.1364 (6)	0.64722 (19)	0.1112 (9)	
H14A	0.4632	−0.2430	0.6065	0.133*	
H14B	0.4734	−0.1797	0.6974	0.133*	
C15	0.3454 (2)	−0.1625 (6)	0.6560 (2)	0.1342 (12)	
H15A	0.3320	−0.3183	0.6697	0.201*	
H15B	0.3152	−0.1235	0.6064	0.201*	
H15C	0.3255	−0.0627	0.6977	0.201*	

### Atomic displacement parameters (Å^2^)

**Table:**

	*U*^11^	*U*^22^	*U*^33^	*U*^12^	*U*^13^	*U*^23^
O1	0.0772 (8)	0.0636 (8)	0.0520 (8)	0.0168 (6)	0.0057 (6)	0.0123 (6)
N1	0.0628 (9)	0.0464 (8)	0.0552 (9)	0.0099 (7)	0.0081 (7)	−0.0002 (7)
N2	0.0616 (9)	0.0464 (8)	0.0471 (8)	0.0075 (7)	0.0023 (6)	0.0000 (6)
C2	0.0494 (9)	0.0448 (9)	0.0518 (9)	−0.0031 (7)	0.0033 (7)	0.0014 (8)
C1	0.0515 (9)	0.0476 (10)	0.0538 (10)	0.0006 (8)	0.0054 (7)	0.0034 (8)
C7	0.0569 (10)	0.0452 (9)	0.0474 (9)	−0.0018 (8)	0.0001 (7)	0.0005 (7)
C3	0.0656 (11)	0.0592 (11)	0.0543 (11)	−0.0016 (9)	0.0079 (8)	−0.0064 (9)
C6	0.0856 (14)	0.0506 (11)	0.0615 (12)	0.0135 (10)	−0.0020 (9)	0.0009 (9)
C9	0.0782 (13)	0.0693 (13)	0.0614 (12)	0.0253 (11)	0.0050 (9)	−0.0062 (10)
C4	0.0807 (13)	0.0761 (14)	0.0483 (10)	−0.0052 (11)	0.0001 (9)	0.0033 (10)
C8	0.0783 (13)	0.0520 (11)	0.0536 (10)	0.0100 (9)	0.0000 (9)	−0.0082 (9)
C5	0.0938 (15)	0.0654 (13)	0.0569 (12)	0.0094 (11)	−0.0057 (10)	0.0111 (10)
C10	0.0684 (13)	0.0785 (14)	0.0764 (14)	0.0186 (11)	0.0086 (10)	0.0031 (11)
C11	0.0713 (14)	0.0938 (17)	0.0919 (16)	0.0211 (12)	0.0106 (11)	0.0036 (14)
C12	0.0739 (14)	0.0947 (18)	0.1004 (18)	0.0153 (13)	0.0087 (12)	−0.0010 (15)
C13	0.0711 (14)	0.107 (2)	0.1020 (18)	0.0144 (14)	0.0064 (12)	−0.0025 (16)
C14	0.0817 (17)	0.112 (2)	0.140 (3)	0.0135 (16)	0.0050 (16)	0.0126 (19)
C15	0.088 (2)	0.141 (3)	0.174 (3)	0.0011 (19)	0.0011 (19)	0.013 (2)

### Geometric parameters (Å, º)

**Table:**

O1—C1	1.235 (2)	C8—H8B	0.9700
N1—C1	1.361 (2)	C5—H5	0.9300
N1—C2	1.387 (2)	C10—C11	1.515 (3)
N1—H1	0.8600	C10—H10A	0.9700
N2—C1	1.367 (2)	C10—H10B	0.9700
N2—C7	1.396 (2)	C11—C12	1.507 (4)
N2—C8	1.452 (2)	C11—H11A	0.9700
C2—C3	1.382 (2)	C11—H11B	0.9700
C2—C7	1.391 (2)	C12—C13	1.513 (3)
C7—C6	1.371 (2)	C12—H12B	0.9700
C3—C4	1.381 (3)	C12—H12A	0.9700
C3—H3	0.9300	C13—C14	1.511 (4)
C6—C5	1.382 (3)	C13—H13A	0.9700
C6—H6	0.9300	C13—H13B	0.9700
C9—C10	1.507 (3)	C14—C15	1.484 (4)
C9—C8	1.511 (3)	C14—H14A	0.9700
C9—H9A	0.9700	C14—H14B	0.9700
C9—H9B	0.9700	C15—H15A	0.9600
C4—C5	1.378 (3)	C15—H15B	0.9600
C4—H4	0.9300	C15—H15C	0.9600
C8—H8A	0.9700		
			
C1—N1—C2	110.42 (14)	C6—C5—H5	119.2
C1—N1—H1	124.8	C9—C10—C11	113.20 (18)
C2—N1—H1	124.8	C9—C10—H10A	108.9
C1—N2—C7	109.50 (14)	C11—C10—H10A	108.9
C1—N2—C8	124.01 (14)	C9—C10—H10B	108.9
C7—N2—C8	126.49 (14)	C11—C10—H10B	108.9
C3—C2—N1	132.54 (16)	H10A—C10—H10B	107.8
C3—C2—C7	120.98 (16)	C12—C11—C10	114.20 (18)
N1—C2—C7	106.48 (14)	C12—C11—H11A	108.7
O1—C1—N1	127.41 (16)	C10—C11—H11A	108.7
O1—C1—N2	125.84 (16)	C12—C11—H11B	108.7
N1—C1—N2	106.75 (14)	C10—C11—H11B	108.7
C6—C7—C2	121.62 (16)	H11A—C11—H11B	107.6
C6—C7—N2	131.52 (16)	C11—C12—C13	115.3 (2)
C2—C7—N2	106.85 (14)	C11—C12—H12B	108.5
C4—C3—C2	117.20 (17)	C13—C12—H12B	108.5
C4—C3—H3	121.4	C11—C12—H12A	108.5
C2—C3—H3	121.4	C13—C12—H12A	108.5
C7—C6—C5	117.19 (18)	H12B—C12—H12A	107.5
C7—C6—H6	121.4	C14—C13—C12	114.0 (2)
C5—C6—H6	121.4	C14—C13—H13A	108.7
C10—C9—C8	114.54 (16)	C12—C13—H13A	108.7
C10—C9—H9A	108.6	C14—C13—H13B	108.7
C8—C9—H9A	108.6	C12—C13—H13B	108.7
C10—C9—H9B	108.6	H13A—C13—H13B	107.6
C8—C9—H9B	108.6	C15—C14—C13	115.6 (2)
H9A—C9—H9B	107.6	C15—C14—H14A	108.4
C5—C4—C3	121.48 (18)	C13—C14—H14A	108.4
C5—C4—H4	119.3	C15—C14—H14B	108.4
C3—C4—H4	119.3	C13—C14—H14B	108.4
N2—C8—C9	113.18 (15)	H14A—C14—H14B	107.4
N2—C8—H8A	108.9	C14—C15—H15A	109.5
C9—C8—H8A	108.9	C14—C15—H15B	109.5
N2—C8—H8B	108.9	H15A—C15—H15B	109.5
C9—C8—H8B	108.9	C14—C15—H15C	109.5
H8A—C8—H8B	107.8	H15A—C15—H15C	109.5
C4—C5—C6	121.53 (19)	H15B—C15—H15C	109.5
C4—C5—H5	119.2		
			
C1—N1—C2—C3	178.89 (18)	N1—C2—C3—C4	−179.26 (18)
C1—N1—C2—C7	−0.48 (18)	C7—C2—C3—C4	0.0 (3)
C2—N1—C1—O1	−178.99 (16)	C2—C7—C6—C5	0.8 (3)
C2—N1—C1—N2	0.88 (18)	N2—C7—C6—C5	179.37 (18)
C7—N2—C1—O1	178.93 (16)	C2—C3—C4—C5	0.5 (3)
C8—N2—C1—O1	−1.0 (3)	C1—N2—C8—C9	−105.19 (19)
C7—N2—C1—N1	−0.95 (18)	C7—N2—C8—C9	74.9 (2)
C8—N2—C1—N1	179.15 (15)	C10—C9—C8—N2	64.2 (2)
C3—C2—C7—C6	−0.7 (3)	C3—C4—C5—C6	−0.3 (3)
N1—C2—C7—C6	178.77 (17)	C7—C6—C5—C4	−0.3 (3)
C3—C2—C7—N2	−179.57 (15)	C8—C9—C10—C11	179.88 (17)
N1—C2—C7—N2	−0.11 (17)	C9—C10—C11—C12	177.1 (2)
C1—N2—C7—C6	−178.07 (19)	C10—C11—C12—C13	−179.1 (2)
C8—N2—C7—C6	1.8 (3)	C11—C12—C13—C14	179.6 (2)
C1—N2—C7—C2	0.66 (18)	C12—C13—C14—C15	−179.7 (3)
C8—N2—C7—C2	−179.44 (15)		

### Hydrogen-bond geometry (Å, º)

**Table:**

*D*—H···*A*	*D*—H	H···*A*	*D*···*A*	*D*—H···*A*
N1—H1···O1^i^	0.86	2.01	2.8257 (19)	159
C4—H4···O1^ii^	0.93	2.52	3.312 (2)	144

Symmetry codes: (i) −*x*+2, −*y*, −*z*+1; (ii) *x*, −*y*+1/2, *z*−1/2.

## References

[bb1] Bruker (2005). *APEX2* and *SAINT* Bruker AXS Inc., Madison, Wisconsin, USA.

[bb2] Farrugia, L. J. (1997). *J. Appl. Cryst.* **30**, 565.

[bb3] Kandri Rodi, Y., Ouazzani Chahdi, F., Essassi, E. M., Luis, S. V., Bolte, M. & El Ammari, L. (2011). *Acta Cryst.* E**67**, o3340–o3341. 10.1107/S1600536811047829 PMC3238987 22199836

[bb4] Ouzidan, Y., Kandri Rodi, Y., Butcher, R. J., Essassi, E. M. & El Ammari, L. (2011). *Acta Cryst.* E**67**, o283. 10.1107/S1600536810054164 PMC3051596 21522975

[bb5] Sheldrick, G. M. (2008). *Acta Cryst.* A**64**, 112–122. 10.1107/S0108767307043930 18156677

[bb6] Soderlind, K. J., Gorodetsky, B., Singh, A. K., Bachur, N., Miller, G. G. & Lown, J. W. (1999). *Anti-Cancer Drug Des.* **14**, 19–36. 10363025

[bb7] Spek, A. L. (2009). *Acta Cryst.* D**65**, 148–155. 10.1107/S090744490804362X PMC2631630 19171970

[bb8] Westrip, S. P. (2010). *J. Appl. Cryst.* **43**, 920–925.

